# Influence of age on voice quality after transoral CO_2_ laser microsurgery

**DOI:** 10.1186/s40463-023-00664-3

**Published:** 2023-09-12

**Authors:** Jerome R. Lechien, Stephane Hans, Lise Crevier-Buchman

**Affiliations:** 1 Department of Otorhinolaryngology and Head and Neck Surgery, Foch Hospital, School of Medicine, UFR Simone Veil, Université Versailles Saint-Quentin-en-Yvelines (Paris Saclay University), Paris, France; 2 Department of Otolaryngology-Head & Neck Surgery, CHU de Bruxelles, CHU Saint-Pierre, Brussels, Belgium; 3 Department of Otolaryngology, Polyclinic of Poitiers, Elsan Hospital, Poitiers, France; 4 Faculty of Medicine, Department of Human Anatomy and Experimental Oncology, UMONS Research Institute for Health Sciences and Technology, University of Mons (UMons), Mons, Belgium; 5 Phonetics and Phonology Lab, CNRS UMR7018, Univ. Sorbonne University, Paris, France

**Keywords:** Larynx, Laryngeal, Cancer, Laser, CO2, Cordectomy, Microsurgery, Voice, Otolaryngology, Head neck, Laryngology

## Abstract

**Objective:**

To study the post-operative evolution of voice quality of patients treated by transoral CO_2_ laser microsurgery (TLM) according to the age.

**Methods:**

Patients treated by type I to VI TLM and post-operative speech therapy were prospectively recruited from our hospital. The voice quality was assessed pre-, 1-, 3- 6- and 12-month posttreatment with voice handicap index (VHI), dysphonia, roughness, breathiness, asthenia, strain (GRBAS), maximal phonation time (MPT), F0, F0 standard deviation (STD), percent jitter, percent shimmer, noise-to-harmonic ratio (NHR), vocal fold vibration assessment and speech fluency. Evolution of voice outcomes was analyzed considering age of patients (< 60 vs. ≥ 60 years).

**Results:**

Seventy-five patients completed the evaluations. Thirty-four and forty-one patients were < 60 or ≥ 60 yo, respectively. Subjective and objective voice parameters (VHI, G, R, B), jitter and fluency significantly improved from 1- to 6-month post-TLM in < 60 yo individuals. The voice parameters improved 12-month post-TLM in the ≥ 60 yo group at the exception of VHI that improved 3-month post-TLM. There were positive associations between age and 12-month NHR, G and A parameters.

**Conclusion:**

The post-operative evolution of voice quality parameters may vary between patients according to the age. Preoperative VHI is predictive of 12-month subjective and objective voice outcomes.

**Graphical abstract:**

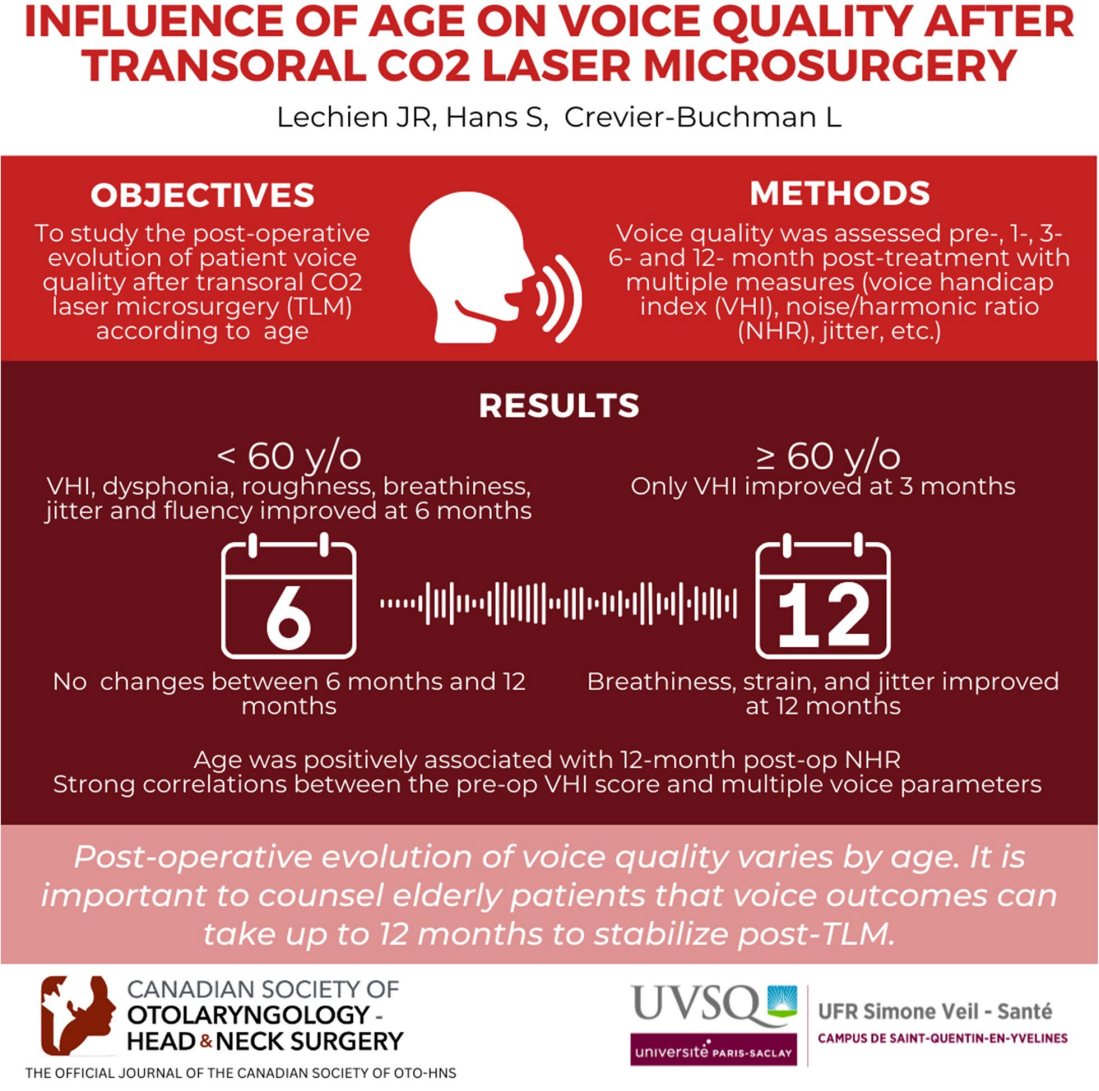

## Introduction

Transoral CO_2_ laser microsurgery (TLM) is an important therapeutic option for primary early-stage vocal fold carcinoma [1, 2]. TLM reported comparable oncological and survival outcomes than radiation [2]. To date, there were many studies investigating voice quality outcome evolution after TLM [3–9]. Most studies suggested that voice quality outcomes may reach stability 6-month post-TLM but the heterogeneity between studies about the tumor stages of patients, the types of TLM, the use of speech therapy, the voice quality outcomes and the time of evaluations may limit the draw of clear conclusion [3–9]. Age of patient was suspected to be an additional factor influencing the vocal cord healing, voice recovery and, therefore, the post-operative evolution of voice measurements [10]. The consideration of aging voice in the post-TLM voice recovery makes particularly sense regarding the recent literature supporting high prevalence of presbyphonia in outpatients consulting a tertiary laryngology consultation [11, 12]. Indeed, Maxwell et al. recently reported that the prevalence of presbylarynx reached 52.4% of North American population [11], while Takano et al. reported that the number of elderly patients with vocal fold atrophy substantially increased with age, especially in male patients with 65% prevalence [12]. Thus, presbyphonia may be an additional factor of post-TLM dysphonia to other age-related factors that may influence voice outcomes, e.g. reduced lung capacity, differences in tissue healing and vocal cord composition [9, 10].

The aim of this study was to investigate the 12-month evolution of voice quality measurements after TLM according to the age of patients (< 60 *versus* ≥ 60 years).

## Methods

### Patients and setting

Patients with early-stage vocal fold carcinoma were recruited from the Department of Otolaryngology-Head & Neck Surgery of the Georges Pompidou European Hospital (Paris, France). To be included, native French-speaker patients had primary early-stage vocal cord carcinoma (cTis, cT1a, cT1b or cT2). Patients with laryngeal surgery, trauma or radiation history, as well as those who required re-operation for another laryngeal lesion within the 12-month follow-up period were excluded. The local ethics committee approved the study protocol, and the informed consent was obtained for all patients (AP-HP Review Board, Hopital Européen George Pompidou: 201602).

### Transoral CO_2_ laser microsurgery

**Fig. 1:**
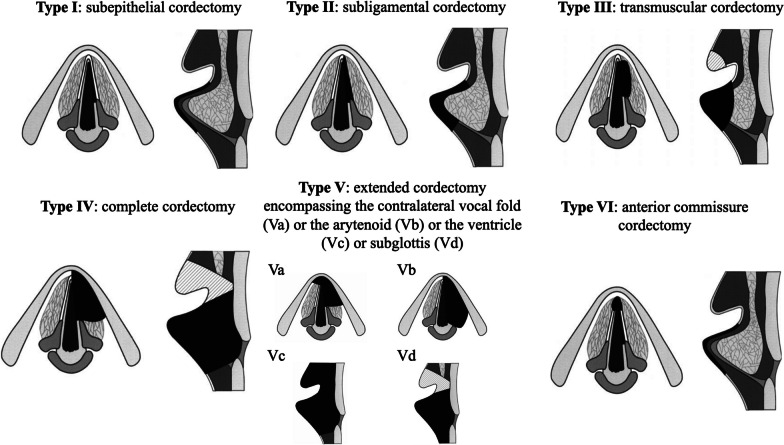
The European Laryngological Society classification of endoscopic cordectomies. This classification was published by Remacle et al. in 2000^11^ and 2007.^12^

The preoperative oncological check-up included stroboscopic examination, endoscopy under general anesthesia and chest, head and neck tomodensitometry. Regarding the local oncological board, the senior laryngologist (SH) proposed to patients the following treatments considering tumor size, location, and stage: TLM (cordectomy) or radiation [13]. When patient chosen surgery, surgeon carried out the most appropriate TLM procedure according to the Proposal for Revision of the European Laryngological Society Classification (Fig. 1) [14]: subepithelial (type I), subligamental (type II), transmuscular (type III), total vocal cord excision (type IV), extended (type V) and anterior bilateral cordectomy and commissurectomy (type VI). The types Va, b, c, and d are extended cordectomies encompassing the contralateral vocal fold (a), the arytenoid (b), ventricle (c) or subglottis (d), respectively. In some cases, the type III-IV resection involved partial resection of the ventricular fold to have an adequate exposure of the entire vocal fold (hatched area on Fig. 1). The surgical steps were described in our previous studies [15, 16].

### Post-operative care and speech therapy

Depending on the TLM type, patients were discharged after 24 to 48-h hospital stay and were instructed to adhere to a voice rest of 3 days. Patients received proton pump inhibitors for a 3-month duration to control the impact of laryngopharyngeal reflux on the vocal fold healing [17]. Patients received 12 sessions of speech therapy (1 time weekly; 3-month duration). The speech therapy started from the second or third post-operative week. The objective was to improve the glottic closure and residual vocal tissue vibration. The steps of the sessions were relaxation and mobilization exercises for the cervicoscapular and orofacial muscles; exercises of control of expiration using voiceless fricative consonants; voiced consonants and vowels; humming sounds; exercises with a straw; and work on the melodic variation. The objective was to avoid deviant compensations increasing the tight or breathy quality of the voice and vocal fatigue.

### Voice analysis

The preoperative, 1-, 3-, 6- and 12-month voice quality evaluations included videolaryngostroboscopy, subjective and objective voice quality assessments. The subjective voice quality was evaluated with the French version of Voice Handicap Index (VHI) [18] and Grade, Roughness, Breathiness, Asthenia and Strain (GRBAS) scale [19], which was retrospectively evaluated by two experienced laryngologists in a blind manner (interrater reliability r_s_ > 0.500) [15]. The perceptual voice quality was assessed on connected speech and reading a phonetically balanced text. The final score of GRBAS was the mean of both laryngologist evaluations. The senior laryngologists (SH & LCB) assessed mucosal wave vibration through a validated visual analog scale ranging from 0 (normal mucosal wave) to 3 (no vocal fold vibration) [20].

Maximum phonation time (MPT) was investigated as aerodynamic measurement. The objective voice quality measurements included the following acoustic parameters: fundamental frequency (F0), standard deviation of F0 (STD), percent jitter, percent shimmer, and noise-to-harmonic ratio (NHR). Acoustic parameters were measured with the Multi-Dimensional Voice Program Kay Elemetrics, Lincoln Park, NJ, USA) on patient sustained /a/ phonation at comfortable intensity and pitch level (3 trials). According to the influence of method of acoustic parameter measurements on the results [21], acoustic parameters were determined for the 3 most stable seconds of the second sustained vowel. The acoustic analyses were performed respecting the European Laryngological Society Statements after a visual control of the period definition on the microphone signal [22].

The MPT consisted of the best duration of the 3 sustained vowel trials. The speech fluency evaluation was based on the reading of a balanced text composed of 169 words and 243 syllables. The final result was reported in number of words per minute.

### Statistical analysis

The statistical analyses were performed with Statistical Package for the Social Sciences for Windows (SPSS version 22.0; IBM Corp, Armonk, NY, USA). Wilcoxon rank test was used to analyze changes in voice outcomes through the 1 to 12-month post-operative period in patient groups (< 60 *versus* ≥ 60 years). The baseline evaluations consisted of the 1-month post-operative voice quality check-up; the 3-, 6- and 12-month voice quality being compared with 1-month post-operative data. Mann–Whitney U and Friedman tests were used for comparisons between groups. The study of outcome association was performed with multivariate analysis. A level of significance of *p* < 0.05 was used.

## Results

**Fig. 2:**
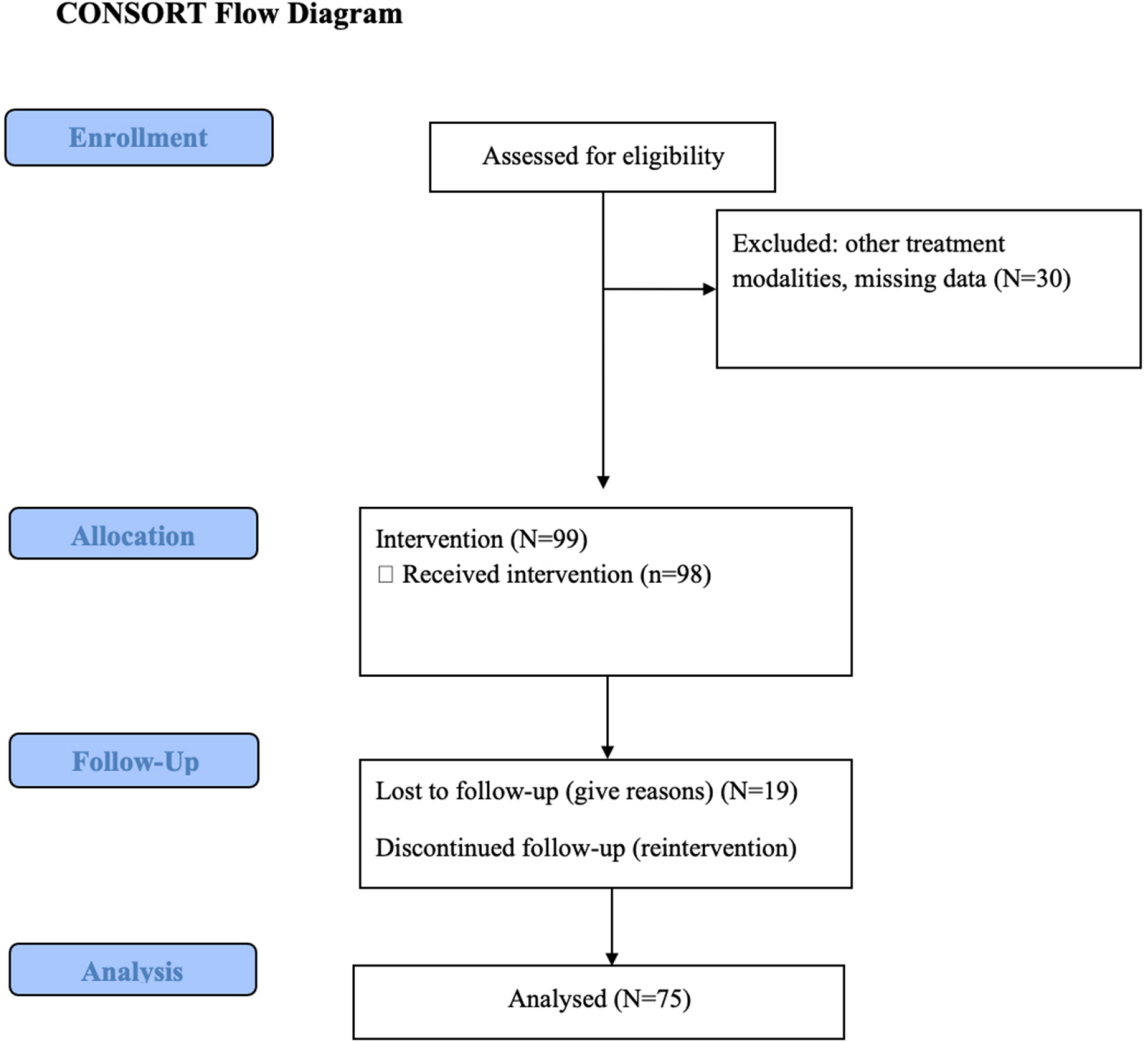
Chart flow

Seventy-five patients completed voice evaluations and speech therapy program (Fig. 2). Thirty-four patients were < 60 yo (range: 28 to 59 yo). Epidemiological features of patients are described in Table 1. Patient groups were comparable regarding gender ratio; smoker and reflux histories; clinical; pathological and treatment outcomes. Granuloma was the main post-operative complication, affecting 4 patients in each group. The overall proportion of TLM types did not differ between groups. Surgical margins were positive in 10% and 14% of < 60 yo and ≥ 60 yo patients, respectively. Patients requiring reintervention for R1 margins were excluded from the study. All patients completed the 12-week speech therapy program.

**Table 1:** Epidemiological and clinical features of patients

Outcomes	< 60 yo (N = 34)	≥ 60 yo (N = 41)	p-value
Gender (M/F)	29/5	36/5	NS
Smoker (pack-year, mean, SD)	26.7 ± 25.3	33.1 ± 28.2	NS
Gastroesophageal reflux (N, %)	5 (15)	10 (24)	NS
Age (ranges)			
25–50 yo	14 (41)	–	–
51–60 yo	20 (59)	–	–
61–70 yo	–	21 (51)	–
71–90 yo	–	20 (49)	–
cTNM			
cTis	14 (41)	13 (32)	NS
cT1a	15 (44)	23 (56)	NS
cT1b	4 (12)	4 (10)	NS
cT2	1 (3)	1 (2)	NS
N0	34 (100)	41 (100)	NS
N +	0 (0)	0 (0)	NS
Margins			
R0	66 (86.8)	41 (74.5)	NS
R1	10 (13.2)	14 (25.5)	NS
Types of cordectomy			
Type I	15 (44)	22 (54)	NS
Type II	5 (15)	6 (15)	
Type III	8 (24)	3 (7)	
Type IV	1 (3)	0 (0)	
Type V	3 (9)	7 (17)	
Type VI	2 (6)	3 (7)	
Complications			
Granuloma	4 (12)	4 (10)	NS
Anterior commissure synechia	0 (0)	1 (2)	NS

*M/F* male/female, *NS* non-significant, *SD* standard deviation, *yo* years old

### Voice analysis

The preoperative voice parameters are reported in Table 2. At baseline, patients < 60 yo reported higher VHI total score than those ≥ 60 yo (p = 0.008).

**Table 2:** Preoperative voice quality parameters

Voice quality outcomes	Preoperative		
	< 60 yo	≥ 60 yo	p-value
VHI	47.2 ± 26.3	32.4 ± 26.9	0.008
GRBAS			
Grade of dysphonia	2.0 ± 0.8	1.9 ± 0.7	NS
Roughness	1.6 ± 0.8	1.7 ± 0.7	NS
Breathiness	1.5 ± 0.6	1.4 ± 0.7	NS
Asthenia	0.1 ± 0.1	0.1 ± 0.3	NS
Strain	1.4 ± 1.0	1.1 ± 0.9	NS
Acoustic measures			
F0 (Hz)	147.8 ± 44.3	157.4 ± 46.0	NS
STD (Hz)	5.6 ± 5.7	6.5 ± 7.8	NS
Jitter (%)	3.1 ± 2.3	3.1 ± 2.4	NS
Shimmer (%)	7.9 ± 4.9	7.9 ± 4.8	NS
NHR	0.2 ± 0.1	0.2 ± 0.1	NS
Maximum phonation time	12.7 ± 5.3	13.1 ± 6.6	NS
Fluency	142.8 ± 17.2	138.4 ± 22.5	NS
Mucosal wave	2.3 ± 1.2	2.2 ± 1.1	NS

*F0* fundamental frequency, *NHR* noise-to-harmonic ratio, *NS* non-significant, *STD* standard deviation of F0, *VHI* voice handicap index, *yo* years old

In the < 60 yo patient group, the improvement of voice parameters occurred at 6-month post-TLM for the following parameters: VHI, G, R, B, jitter and fluency (Table 3). There were no additional changes from 6- to 12-month post-TLM. There were significant positive associations between reflux history and 6-month post-operative jitter (r_s_ = 0.268; p = 0.048) and NHR (r_s_ = 0.354; p = 0.008) values.

**Table 3:** Pre to posttreatment evolution of voice quality in patient group and comparisons

Voice quality outcomes	1-month postoperative		Group	3-month		Group	6-month		Group	12-month		Group
	< 60 yo	≥ 60 yo	p-value	< 60 yo	≥ 60 yo	p-value	< 60 yo	≥ 60 yo	p-value	< 60 yo	≥ 60 yo	p-value
VHI	48.0 ± 41.1	46.3 ± 29.6	NS	33.3 ± 21.9	19.8 ± 20.3	0.007	27.7 ± 22.5	16.3 ± 18.3	0.030	28.0 ± 21.7	12.2 ± 14.5	0.001
GRBAS												
Grade of dysphonia	2.0 ± 0.8	2.2 ± 0.7	NS	1.8 ± 0.9	1.8 ± 0.8	NS	1.4 ± 0.9	1.5 ± 0.8	NS	1.4 ± 0.9	1.5 ± 0.9	NS
Roughness	1.4 ± 0.7	1.5 ± 0.9	NS	1.4 ± 0.9	1.4 ± 0.8	NS	1.3 ± 0.7	1.3 ± 0.7	NS	1.0 ± 0.7	1.3 ± 0.8	NS
Breathiness	1.7 ± 0.9	1.7 ± 0.8	NS	1.3 ± 0.9	1.2 ± 0.9	NS	1.1 ± 0.9	1.0 ± 0.7	NS	1.1 ± 0.8	0.8 ± 0.7	NS
Asthenia	0.1 ± 0.2	0.1 ± 0.5	NS	0.2 ± 0.5	0.1 ± 0.2	NS	0.1 ± 0.1	0.1 ± 0.1	NS	0.1 ± 0.1	0.1 ± 0.2	NS
Strain	1.1 ± 0.9	1.1 ± 0.9	NS	0.8 ± 0.8	1.0 ± 0.9	NS	0.8 ± 0.9	0.8 ± 0.8	NS	0.8 ± 0.9	0.6 ± 0.7	NS
Acoustic measures												
F0 (Hz)	154.4 ± 52.8	171.5 ± 43.8	NS	158.2 ± 51.5	168.0 ± 44.0	NS	156.7 ± 44.4	155.9 ± 38.9	NS	156.2 ± 49.4	154.8 ± 38.0	NS
STD (Hz)	5.4 ± 4.7	9.8 ± 12.6	NS	6.8 ± 9.6	6.5 ± 5.7	NS	5.9 ± 14.5	4.0 ± 2.4	NS	7.4 ± 13.8	4.3 ± 4.2	NS
Jitter (%)	3.6 ± 4.0	3.7 ± 3.1	NS	3.5 ± 3.2	2.8 ± 2.4	NS	3.0 ± 3.9	2.7 ± 2.6	NS	2.7 ± 2.9	2.7 ± 2.8	NS
Shimmer (%)	7.0 ± 4.0	8.2 ± 5.7	NS	7.4 ± 4.6	6.7 ± 4.6	NS	6.2 ± 3.8	7.3 ± 5.4	NS	6.7 ± 4.4	7.7 ± 6.3	NS
NHR	0.2 ± 0.1	0.2 ± 0.1	NS	0.2 ± 0.1	0.2 ± 0.1	NS	0.2 ± 0.1	0.2 ± 0.1	NS	0.2 ± 0.1	0.2 ± 0.1	NS
Maximum phonation time	9.8 ± 4.6	11.6 ± 5.8	NS	9.9 ± 5.3	11.5 ± 7.5	NS	10.9 ± 5.4	10.5 ± 5.1	NS	11.2 ± 5.6	11.2 ± 6.0	NS
Fluency	141.0 ± 19.2	138.2 ± 22.8	NS	143.5 ± 19.0	136.9 ± 22.9	NS	149.0 ± 19.0	139.5 ± 19.8	NS	147.7 ± 19.7	140.1 ± 19.1	NS
Mucosal wave	1.5 ± 2.1	2.3 ± 1.5	NS	2.7 ± 0.5	1.7 ± 1.1	NS	2.1 ± 0.9	1.6 ± 0.9	NS	1.6 ± 1.1	1.6 ± 0.9	NS

*F0* fundamental frequency, *M/F* male/female, *NHR* noise-to-harmonic ratio, *SD* standard deviation, *STD* standard deviation of F0, *VHI* voice handicap index, *yo* years old

In the ≥ 60 yo group, VHI significantly reduced at 3-month post-TLM, while breathiness, strain, STD and jitter are the voice parameter that significantly reduced post-TLM (12-month). The group comparison reported that the 3-, 6- and 12-month VHI scores were significantly lower in ≥ 60 yo compared with < 60 yo group (Table 3).

In sum, in the ≥ 60 yo group, VHI improved significantly at 3-month post-TLM, while most other parameters (breathiness, strain, STD and jitter) did not improve until 12 months post-TLM. In the < 60 yo group, most parameters improved at 6-month post-treatment.

### Association study

The multivariate analysis reported significant negative associations between age and the following outcomes: 3-month post-operative VHI (r_s_ = − 0.283; p = 0.020); 12-month post-operative VHI (r_s_ = − 0.313; p = 0.014) and word count (r_s_ = − 0.280, p = 0.031). Age was positively associated with 12-month post-operative NHR (r_s_ = 0.257; p = 0.044), grade of dysphonia (r_s_ = 0.351; p = 0.005) and asthenia (r_s_ = 0.266; p = 0.036). The post-operative consumption of tobacco did not significantly impact the voice quality parameters.

Baseline VHI was an indicator of the 1-month post-operative VHI score (r_s_ = 0.847; p = 0.016) and word count (r_s_ = − 0.402; p = 0.008); the 3-month post-operative VHI (r_s_ = 0.528; p = 0.001), jitter (r_s_ = 0.328; p = 0.010), shimmer (r_s_ = 0.408; p = 0.001), NHR (r_s_ = 0.350, p = 0.006) and the 6-month post-operative VHI (r_s_ = 0.485; p = 0.001). There were strong correlations between the preoperative VHI score and the following 12-month subjective and objective voice parameters: VHI (r_s_ = 0.530; p = 0.001); jitter (r_s_ = 0.376; p = 0.004); shimmer (r_s_ = 0.371; p = 0.004); NHR (r_s_ = 0.283; p = 0.031); grade of dysphonia (r_s_ = 0.343; p = 0.008); breathiness (r_s_ = 0.400; p = 0.002); strain (r_s_ = 0.374; p = 0.004); and word count (r_s_ = − 0.285; p = 0.033).

## Discussion

The identification of useful voice quality outcomes is an important issue for the follow-up of TLM patients benefiting from speech therapy. Subjective and objective voice parameters may provide patient quality of life information and recovery findings to the laryngologist and the speech therapist who may assess the post-operative voice quality evolution.

The primary finding of the present study is the observation of an influence of age on post-operative voice parameter evolution. Indeed, our data supported that most voice quality parameters significantly improved earlier (6-month post-TLM) in younger patients compared with elderly individuals who had the most important improvements at 12-month post-TLM. The stronger correlation between age, NHR, G and A parameters at 12-month post-TLM is an additional observation supporting this primary finding. The improvement and stabilization of some vocie parameters at 6-month post-TLM corroborate the results of the study of Hendriksma et al*.* who reported a 6-month improvement of VHI in cT1-2 TLM patients [23]. In the same vein, Chu et al. observed that the mean airflow rate, VHI, astheny and strain parameters improved after 6 months in 25 patients benefiting from type I-II cordectomies [4]. The influence of age on voice parameter evolution was supported by the study of Lane et al*.* who observed that age and tumor stage were important factors in the voice improvement of patients benefiting from TLM for cT1 or cT2 cancer. Precisely, these authors demonstrated that younger patients reported better voice quality in the months following the TLM procedure [4]. The decreased pulmonary function in long-lasting smokers affected by COPD is another possible explanation for slower recovery of vocal functions after TLM of elderly individuals. Note that the group of patients < 60 years had baseline higher VHI than the group of elderly patients, whereas the VHI score improved earlier in elderly than younger group. This significant difference may be attributed to the lowest voice use in daily life of elderly patients who were mostly retired. Thus, it has been suggested that patient-perception of voice impairment may be influenced by occupational activities [39]. Elderly patients may have a lower impact of disease on voice quality perception (VHI) at baseline than younger individuals and, moreover, better postoperative satisfaction than younger patients in terms of voice quality recovery.

The voice quality measurements are influenced by the vibratory properties of tissues, and the patient ability to develop compensatory mechanisms in the voice production. According to basic science and clinical studies, elderly patients may be characterized by less effective vocal fold healing and regeneration process than younger individuals, which may support the findings of the present study [24–26]. Another determinant factor in the voice recovery is the adherence to speech therapy [27]. In the present study, all patients adhered adequately to speech therapy program with experienced speech therapists, which may reduce the risk of evaluation bias. However, the access to speech therapy may be limited in some world regions or country provinces, which limits the generalizability of these results.

Laryngopharyngeal reflux is known to significantly impact the vocal fold defense mechanisms and biomechanical properties, leading to voice quality impairments [28, 29]. In our study, we observed that the presence of reflux was associated with higher 6-month post-operative jitter and NHR values that may indirectly suggest a potential negative role of reflux in the vocal tissue healing or functioning. Laryngeal granuloma is an usual post-operative complication related to tissue injury and healing disorders [30]. Eight patients (10.7%) had post-operative granuloma over the follow-up period, which corroborates the data of the literature [30, 31]. Although that PPIs do not totally protect against the reflux effect (intracellular pepsin activity) 26, the systematic introduction of post-operative PPI treatment, alginate and speech therapy probably limited the development of tissue inflammation and related granuloma.

Interestingly, we observed that preoperative VHI had a predictive value on 12-month objective and subjective voice parameters (i.e. VHI, jitter, shimmer, NHR, G, B, S, and fluency). VHI highlights the impact of voice disorder on the patient quality-of-life and it is important to evaluate the patient perception of its ability to communicate and manage the dysphonia. Several explanations may support the predictive value of VHI. First, the voice use may vary from one patient to another. Patients with an important use of voice in their daily life may report higher VHI in case of laryngeal disease [32]. Thus, as reported by Makeieff et al., both preoperative and post-operative VHI scores may be influenced by the patient lifestyle; those being significantly impacted by the cancer or post-operative dysphonia reporting higher scores at these two times [32]. The high variability of VHI score in TLM patients was supported in the study of Lee et al*.* who reported that patient-perceived voice function improved to normal after treatment in only 62.5% of patients [33]. Second, it is possible that patients with more extended carcinoma and related more aggressive TLM reported higher baseline and post-operative VHI scores due to tumor and treatment features. This hypothesis was supported by Al-Mamgani et al. and Peretti et al. who observed relationship between tumor stage and VHI scores [34, 35].

Aerodynamic and acoustic measurements are useful parameters to study the vibratory process of the vocal folds. We observed that STD and jitter values improved throughout the 12-month follow-up period in both patient groups. The improvement of acoustic parameters may highlight the recovery of the vibratory process of the vocal folds that may be related to the vocal fold tissue healing over time and the development of compensation mechanisms with the speech therapy.

The low prevalence of postoperative laryngeal synechia in types V and VI cordectomies may be attributed to the systematic use of antireflux treatment in postoperative period. Indeed, laryngopharyngeal reflux (acidic pepsin) has been found to negatively influence the vocal cord healing after surgery [36, 37].

The primary limitations of the present study were the low number of patients and the consideration of several types of TLM. The voice quality evolution may differ from one TLM to another according to the post-operative anatomical defects [16, 38]. Another limit is the evaluation of GERD and not LPR. Although the proportion of TLM types did not differ between groups, future large cohort studies are needed to study the impact of age on post-operative voice parameters according to the TLM type. Another limitation involved the moderate number of patients who were excluded for missing data. It was difficult to know the reason of the lack of follow-up of some of them, which may imply some degree of bias in the post-operative voice quality findings.

## Conclusion

The post-operative evolution of voice quality parameters may vary between patients according to the age. The improvement of post-operative voice quality occurred after 6 months in younger patients, whereas elderly individuals reported 12-month overall voice parameter improvement. Preoperative VHI is predictive of 12-month subjective and objective voice outcomes. Our findings support that it is important to counsel elderly patients that voice outcomes can take up to 12 months to stabilize post-TLM.

## Data Availability

Data are available on request.

## Abbreviations

F0: Fundamental frequency
GRBAS: Grade, roughness, breathiness, asthenia and strain
TLM: Transoral CO_2_ laser microsurgery
STD: Standard deviation of F0
VHI: Voice handicap index.
MPT: Maximum phonation time
NHR: Noise-to-harmonic ratio
